# Computational Aspects of Carbon and Boron Nanotubes

**DOI:** 10.3390/molecules15128709

**Published:** 2010-11-30

**Authors:** Paul Manuel

**Affiliations:** Department of Information Science, Kuwait University, Kuwait; E-Mail: p.manuel@ku.edu.kw

**Keywords:** carbon nanotubes, boron nanotubes, maximum independent set, perfect matching, matching ratio, broadcasting algorithm

## Abstract

Carbon hexagonal nanotubes, boron triangular nanotubes and boron α-nanotubes are a few popular nano structures. Computational researchers look at these structures as graphs where each atom is a node and an atomic bond is an edge. While researchers are discussing the differences among the three nanotubes, we identify the topological and structural similarities among them. We show that the three nanotubes have the same maximum independent set and their matching ratios are independent of the number of columns. In addition, we illustrate that they also have similar underlying broadcasting spanning tree and identical communication behavior.

## 1. Introduction

**Figure 1:**
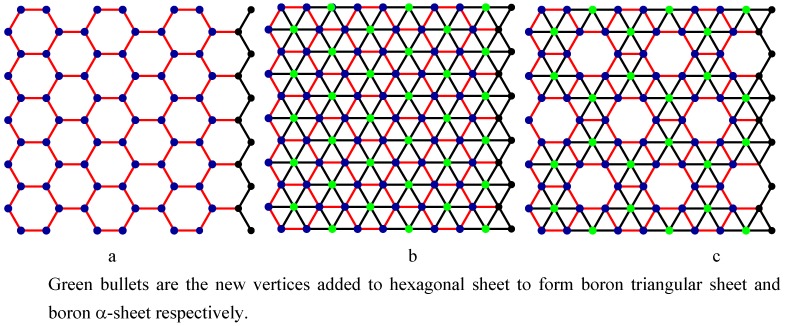
a. Carbon hexagonal sheet (1991); b. Boron triangular sheet (2004); c. Boron α-sheet (2008).

Nanotechnology is defined as the study and use of structures between 1 nanometer and 100 nanometers in size. Nanotechnology creates many new materials and devices with a wide range of applications in medicine, electronics, and computer. Nanotechnology is expected to revolutionize the 21^st^ century as space, entertainment and communication technology revolutionized the 20^th^ century. It involves different structures of nanotubes. The most significant nano structures are carbon nanotubes, boron triangular nanotubes and boron α-nanotubes. See Figure 1a,b,c. Nanotubes are three dimensional cylindrical structures formed out of the two dimensional sheets. 

Carbon nanotubes consist of carbon atoms bonded into a tube shape where carbon atoms are located at apexes of regular hexagons on two-dimensional surfaces. Carbon nanotubes are extremely strong, probably one of the strongest materials that is even theoretically possible. Recently, carbon nanotubes have been proposed as a building material for armor so strong that bullets bounce right off it. The use of carbon nanotubes will allow the computing industry to create computers more powerful than those which can be fabricated via the conventional method of photolithography [1]. Researchers at NASA are combining carbon nanotubes with other materials into composites that can be used to build lightweight spacecraft [2]. Carbon nanotubes are everywhere such as building and textile materials, computers, and lightweight spacecraft. New functionalized nanotubes applications will come onto the market in the next few years that will greatly increase global revenues to $2.7 billion plus by 2015; driven mainly by the needs of the electronics and data storage, defense, energy, aerospace, and automotive industries [3]. 

The recent discovery of pure boron triangular nanotubes challenges the monopoly of carbon. The first boron triangular nanotubes were created in 2004 and are formed from a triangular sheet [4,5,6]. Figure 1b shows a boron triangular sheet. A *boron triangular sheet* is obtained from a hexagonal sheet by adding an extra atom to the centre of *each* hexagon. Scientists believe that boron triangular nanotubes are better than carbon hexagonal nanotubes [4,7,8]. Peter Miller [6] states: “if 2007 was the year of the carbon nanotube, it looks like 2008 could be the year for boron nanotubes to shine”. Sohrab Ismail-Beigi [9] of Yale University speculates “If a superconducting nano computer is ever built, it might have boron wiring”. Lately scientists have justified this speculation by discovering the world’s smallest superconductor using nano scale molecular superconducting boron wires [10]. 

Most recently, researchers [7,11,12] have fabricated special boron sheet from a hexagonal sheet by adding an atom to the centre of *certain* hexagons. They have designed the sheet by generating a mixture of hexagons and triangles. This special boron sheet is called *boron α-sheet* (see Figure 1c). Boron α-sheet involves 1/9 of the atoms missing from the original boron triangular sheet [7,11]. Researchers claim that this is the best configuration and the most energetically stable known theoretical structure for a boron nanotube. Going one step ahead, some researchers [7,8,11,12,13] call for reconsideration of the literature on boron triangular sheets, nanotubes, and clusters. 

While researchers are debating on the differences of these structures, this paper identifies common structural and topological properties of the nanotubes. Tian *et al.* [13] have investigated the structure, stability, and electronic properties of four types of boron nanotubes. In this paper, we present a comparative study of some topological properties of carbon hexagonal nanotube, boron triangular nanotube and boron α- nanotube. We identify a few topological properties where all the three models behave the same way. We show that the three nanotubes have the same maximum independent set and their matching ratios are independent of the number of columns. We also demonstrate that they also have common underlying broadcasting spanning tree and identical communication behavior. 

## 2. Basic Properties of Nanotubes of Armchair Model

There are different shapes of carbon nanotubes such as armchair, chiral and zigzag [5,14,15,16] based on the rolling of 2D carbon hexagonal sheet. Kunstmann and Quandt hypothesize that zigzag boron nanotubes do not exist [5]. Hence in this paper, we do not discuss zigzag boron nanotubes and focus only on armchair model of carbon nanotubes. Here onwards, a carbon nanotube means an armchair model. 

**Figure 2:**
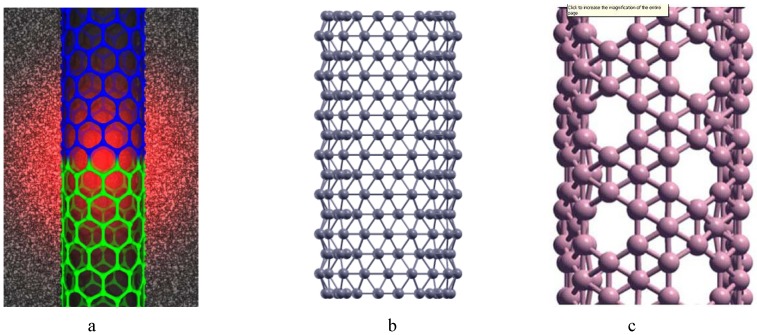
a. Carbon nanotube (1991); b. Boron nanotube (2004); c. Boron α- nanotube (2008).

**Figure 3:**
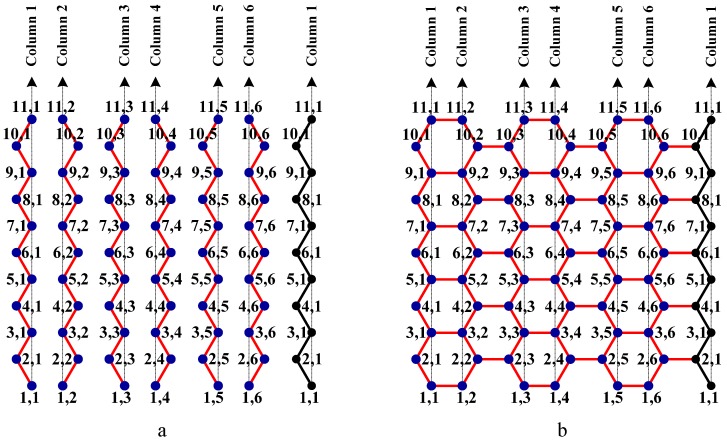
This is an armchair carbon hexagonal nanotube of order 11 × 6. There are six columns and each column has 11 rows of vertices. Each vertex is labeled based on its location with respect to row and column. The first column and the last column of the carbon hexagonal sheet are merged to form a carbon nanotube.

A *carbon hexagonal nanotube of order n*×*m* is a tube obtained from a carbon hexagonal sheet of *n* rows and *m* columns by merging the vertices of last column with the respective vertices of first column (see Figure 3a,b). A *boron triangular nanotube of order n* × *m* is obtained from a hexagonal nanotube of order *n* × *m* by adding a new vertex at the center of each hexagon of the hexagonal nanotube. See Figure 1b and Figure 2b. A *boron α-nanotube of order n* × *m* is obtained from a hexagonal nanotube of order *n* × *m* by adding a new vertex to the centre of some of the hexagons of the hexagonal nanotube (see Figure 1c and Figure 2c).

In order to understand the structural properties of a graph, it is important to have a labeling system to distinguish and identify each vertex and edge of the graph. It is enough to label the vertices of a carbon hexagonal nanotube since a boron triangular nanotube is created by placing an additional vertex at the center of each hexagon of a hexagonal nanotube and a boron α- nanotube is a subgraph of a boron nanotube. A labeling scheme of carbon hexagonal nanotube is given in Figure 3a,b.

**Theorem** **2.1:** A carbon hexagonal nanotube has only odd number of rows and even number of columns.

**Proof:**  Suppose the order of carbon hexagonal nanotube is *n* × *m*. We first prove that *n* is odd. This is true by induction. If there is only one row of hexagons, then *n* = 3. When a row of hexagons is added vertically to the rectangular sheet, *n* is increased by 2. Thus *n* is always odd.

Next we observe that *m* is even. When a carbon hexagonal sheet is rolled to form a nanotube, only the vertices of an odd column are merged with the respective vertices of column 1. Thus *m* is even. 

A complete regular hexagon with six vertices is called *full-hexagon*. An incomplete hexagon with four vertices is called a *half-hexagon*. The first row (last row) of an armchair carbon hexagonal nanotube of order *n* × *m* has *m*/2 number of half-hexagons. See Figure 1a and Figure 3b. Thus we state

**Lemma** **2.2:** There are *m*(*n*–2)/2 number of full hexagons and *m* number of half hexagons in a carbon hexagonal nanotube of order *n* × *m.*

Using Lemma 2.2, it is rather straightforward to compute the number of vertices and edges of carbon hexagonal nanotube, boron triangular nanotube and boron α-nanotube of order *n* × *m* and the proof is left to the reader.

**Theorem 2.3:**

(1) A carbon hexagonal nanotube of order *n* × *m* has *nm* vertices and *m*(3*n*–2)/2 edges.
(2) A boron triangular nanotube of order *n* × *m* has 3*nm/*2 vertices and 3*m*(3*n*–2)/2 edges.
(3) A boron α-nanotube of order *n* × *m* has 4 *nm*/3vertices and *m*(7*n*–4)/2 edges when *n* is a multiple of 3.

## 3. Independent Set of Three Nanotubes

A set S of vertices is *independent* if no two vertices of S are adjacent. The problem of finding a maximum independent set is NP-complete, and still remains so even if we restrict ourselves to the class of planar graphs, cubic planar graphs or triangle free graphs [17]. Tang Jian [18] has designed an O(2^0.304n^) exponential algorithm for solving maximum independent set problem for general graphs. Soares and Stefanes [19] have given a polynomial algorithm to find maximum independent set of convex bipartite graphs. Algorithms are proposed to solve maximum independent set problem of planar graphs [20] and apple-free graphs [21]. In this section we show that the maximum independent set of three nanotubes are the same.

**Theorem** **3.1:** Maximum independent set of carbon hexagonal nanotube, boron triangular nanotube and boron α-nanotube of order *n*×*m* is the same whose size is *nm/*2.

**Proof:**  Let CNT denote a carbon hexagonal nanotube of order *n* × *m*. CNT is bipartite which is two-colorable. Let us color the CNT by red and blue colors. It is easy to verify that the set of red vertices form an independent set of the CNT. Let us now show that the cardinality of any independent set of a CNT of order *n* × *m* does not exceed *nm/*2. There are *m* columns in a CNT of order *n* × *m* and each column is a path. Thus a CNT of order *n*×*m* is partitioned into *m* paths. The cardinality of any independent set of a path of order *n* does not exceed ?*n/*2?. Hence the cardinality of any independent set of a CNT of order *n* × *m* does not exceed *nm/*2. The cardinality of set of red vertices of the CNT is *nm*/2. Hence the set of red vertices is a maximum independent set of the CNT.

**Figure 4:**
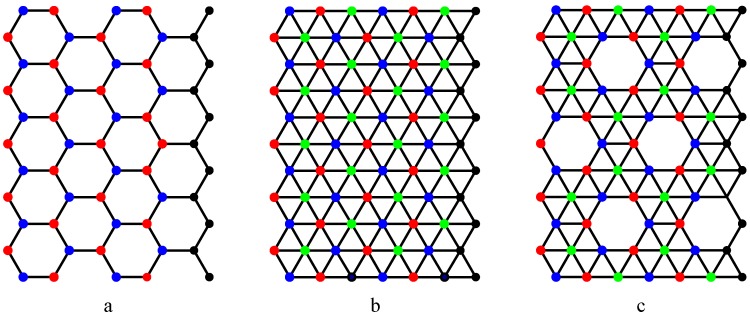
The maximum independent set is the same for all the three nanotubes. The set of red vertices is a maximum independent set of three nanotubes. The set of blue vertices is another maximum independent set.

Next we prove that the set of red nodes is a maximum independent set of boron triangular nanotube. A boron triangular nanotube is obtained by adding a new vertex to the center of each hexagon of carbon hexagonal nanotube. These additional vertices are assigned green color. Thus the vertices of the boron triangular nanotube are partitioned by red, blue and green colors (see Figure 4). A green node cannot be a member of any maximum independent set of the boron triangular nanotube because inclusion of one green node into a maximum independent set leads to the exclusion of three red nodes from the maximum independent set. Thus the set of red nodes is a maximum independent set of the boron triangular nanotube. In the same way, it is easy to show that the set of red nodes (blue nodes) is a maximum independent set of boron α-nanotube. The cardinality of set of red nodes is *nm/*2. 

## 4. Perfect Matching and Matching Ratio

A *matching* is a set of pair wise disjoint edges. It is also called an independent edge set. Matching theory is one of the classical and the most important in combinatorial theory and network flow theory. Let *G*(*V*,*E*) be a graph. A matching *M* is perfect matching if every vertex of *G* is covered by an edge of *M*. A *Kekulé structure* of an aromatic compound coincides with a perfect matching of its carbon skeleton, showing the locations of double bonds in the chemical structure. These structures are named after Friedrich August Kekulé von Stradonitz, who showed that benzene (in graph theoretical terms, a 6-vertex cycle) can be given such a structure. 

**Figure 5:**
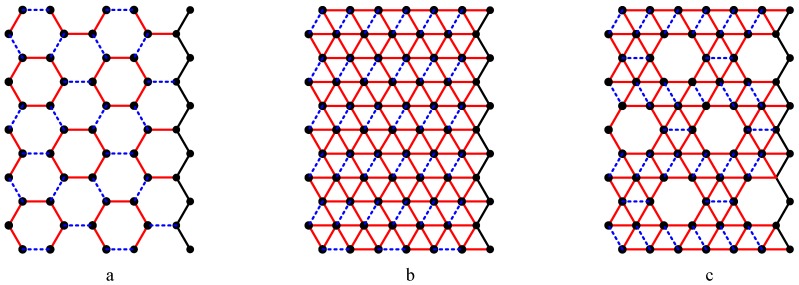
All three nanotubes have perfect matching. The edges of blue dotted lines form a perfect matching.

A benzenoid system is Kekuléan if it has a perfect matching. The Hosoya index *Z*(*G*) of *G* is the total number of matchings in *G*. There is a high correlation between the Hosoya index and the boiling points of the acyclic alkanes. There is huge volume of literature on matchings of hexagonal systems [22,23,24,25]. Our objective of the paper is to identify similar properties of three nanotubes. We just point out that all three nanotubes have perfect matchings and their matching ratios are independent of the number of columns. Figure 5 exhibits a perfect matching for each nanotube.

$$\;Matching\;Ratio\;of\;a\;graph=\frac{Cardinality\;of\;maximum\;matching}{Cardinality\;of\;edge\;set\;of\;the\;graph}\;$$

If a graph has a perfect matching, then:

$$\;Matching\;Ratio\;of\;a\;Graph=\frac{Number\;of\;vertices\;}{2\;Number\;of\;edges\;}\;\;$$

**Theorem** **4.1:** The matching ratios of three nanotubes of order *n*×*m* are independent of the number of columns. The asymptotic matching ratio of carbon hexagonal nanotube = 1/3. The asymptotic matching ratio of boron triangular nanotube = 1/6. The asymptotic matching ratio of carbon hexagonal nanotube = 4/21

**Proof**: Since a carbon nanotube has a perfect matching,

$$\begin{array}{ll} Matching\;ratio\;of\;carbon\;hexagonal\;nanotube & =\frac{Number\;of\;vertices\;}{2\;Number\;of\;edges\;}\\ & =\frac{nm}{m\left(3n-2\right)}\\ & =\frac{n}{\left(3n-2\right)}\to \frac{1}{3}as\;n\to \infty \end{array}$$

$$\begin{array}{ll} Matching\;ratio\;of\;boron\;triangular\;nanotube & =\frac{Number\;of\;vertices\;}{2\;\;Number\;of\;edges\;}\;\\ & =\frac{3nm}{6m\left(3n-2\right)}\\ & =\frac{n}{2\left(3n-2\right)}\to \frac{1}{6}as\;n\to \infty \end{array}$$

$$\begin{array}{ll} Matching\;ratio\;of\;\mathrm{boron}\;-\mathrm{nanotube} & =\frac{Number\;of\;vertices\;}{2\;Number\;of\;edges\;}\;\\ & =\frac{4nm}{3m\left(7n-4\right)}\\ & =\frac{4n}{3\left(7n-4\right)}\to \frac{4}{21}as\;n\to \infty \end{array}$$

The matching ratios of three nanotubes of order *n* × *m* are independent of the number of columns. 

## 5. Broadcasting Problem of Carbon and Boron Nanotubes

The sequence of chemicals which transfer atoms from one to the next to the next to the next is referred to as the *electron transport system*. Horton *et al.* [26] describes an example of a path of electrons released from a molecule to the next to the next to the next by electron carrier NADH (reduced nicotinamide adenine dinucleotide). A similar computational concept is *broadcasting* which is a process of disseminating a message from a source node O to all other nodes of a graph in such a way that in each time unit, an informed vertex can send the message to at most one of its neighbors. In each communication step, a node either transmits or receives. During one time unit (communication step), the message is broadcast from an informed node to uninformed node. The *broadcasting problem* is whether a message can be broadcast in *k* time units. A spanning tree along which the message is broadcast from the source node to all other nodes is called *broadcasting tree* of the network. The broadcasting problem is NP-complete for 3-regular planar graphs and a constant deadline *k* ≥ 2 [27]. This problem is studied almost on all kinds of architectures and systems for example, wireless sensor networks [28], cellular networks of triangular systems [29], heterogeneous tree networks [30], honeycomb networks [31], higher dimensional hexagonal networks [32], mesh architectures [33], star graphs [34], de Bruijn Networks [35], hypercubes [36].

Manuel *et al*. [16] have given an optimal broadcasting algorithm for carbon nanotubes of zigzag model. Interestingly this technique does not work for carbon nanotubes of armchair model. Moreover, our objective is to demonstrate that all three nanotubes have similar broadcasting spanning trees and optimal broadcasting time.

### 5.1. Broadcasting Algorithm for Carbon Hexagonal Nanotubes

The eccentricity *e*(*v*) of a vertex *v* is the greatest distance between *v* and any other vertex of the graph. An eccentric vertex of a vertex *v* is a vertex farthest away from *v*.

**Figure 6:**
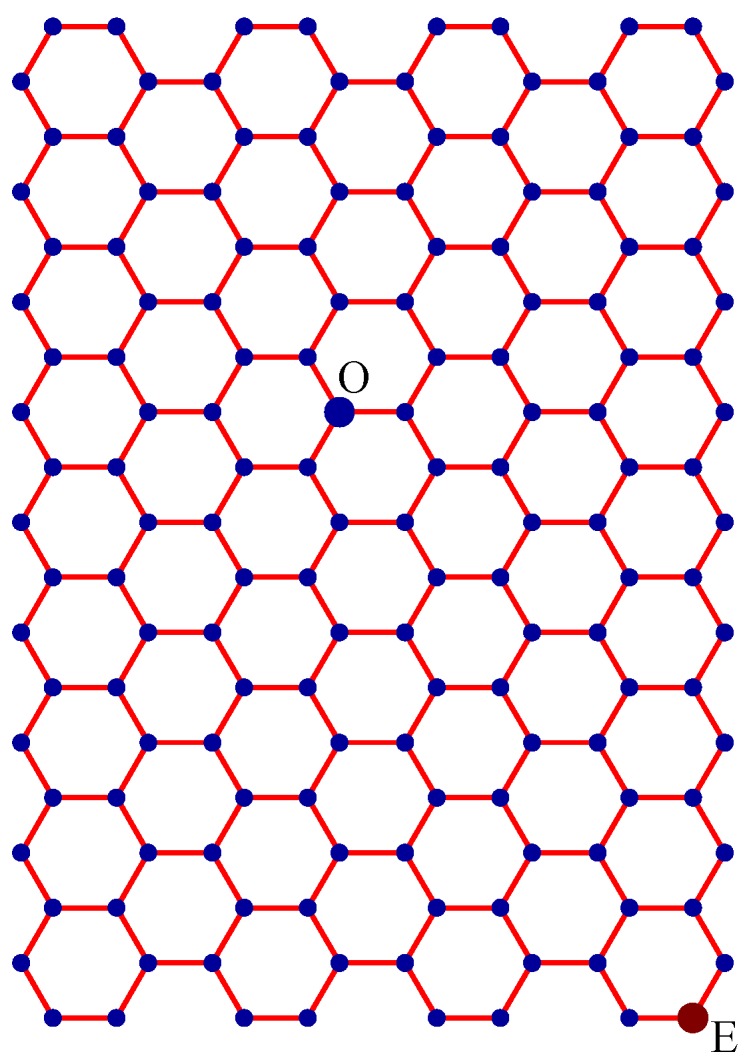
A rectangular sheet of carbon hexagonal nanotube of order 19 × 8. Node O is the source and node E is the eccentric node of O. The wrapping edges between the first column and the last column are not drawn.

**Figure 7:**
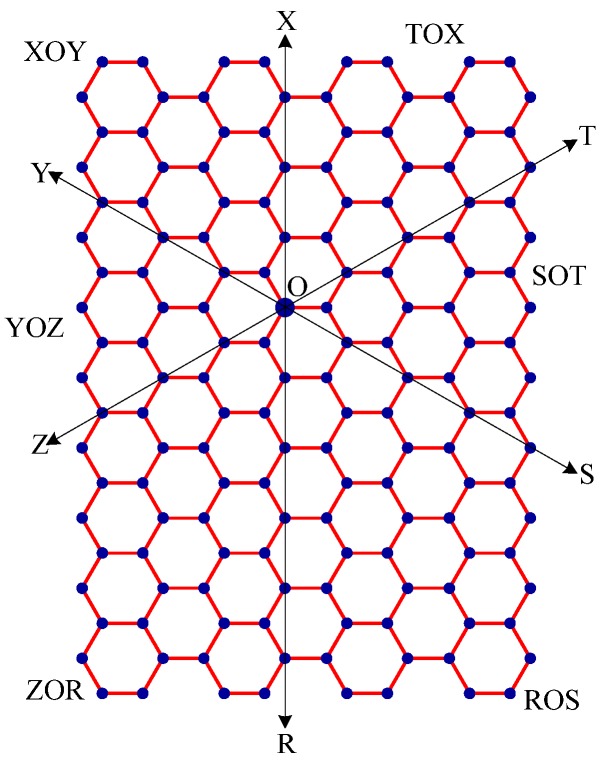
The rectangular sheet is divided into 6 zones: zones ROS, SOT, TOX, XOY, YOZ and ZOR. For example, zone ROS is a subgraph induced by the edges lying between the lines OR and OS.

**Figure 8:**
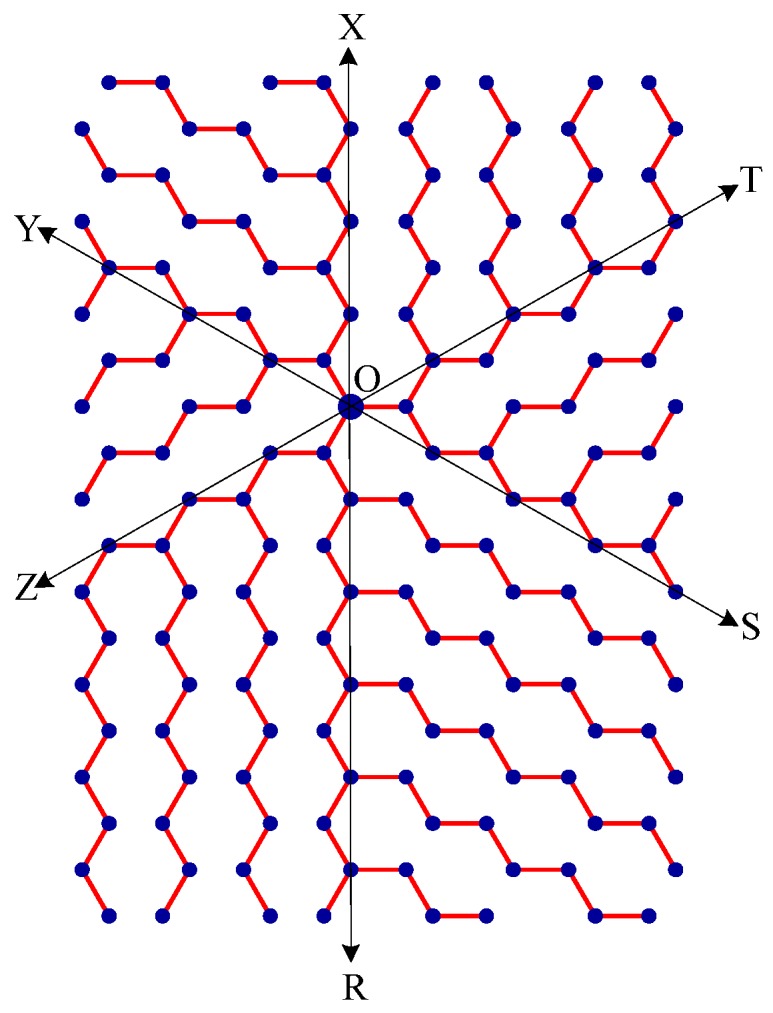
Broadcasting tree of the nanotube. Broadcasting is based on farthest-distance-first protocol.

**Figure 9:**
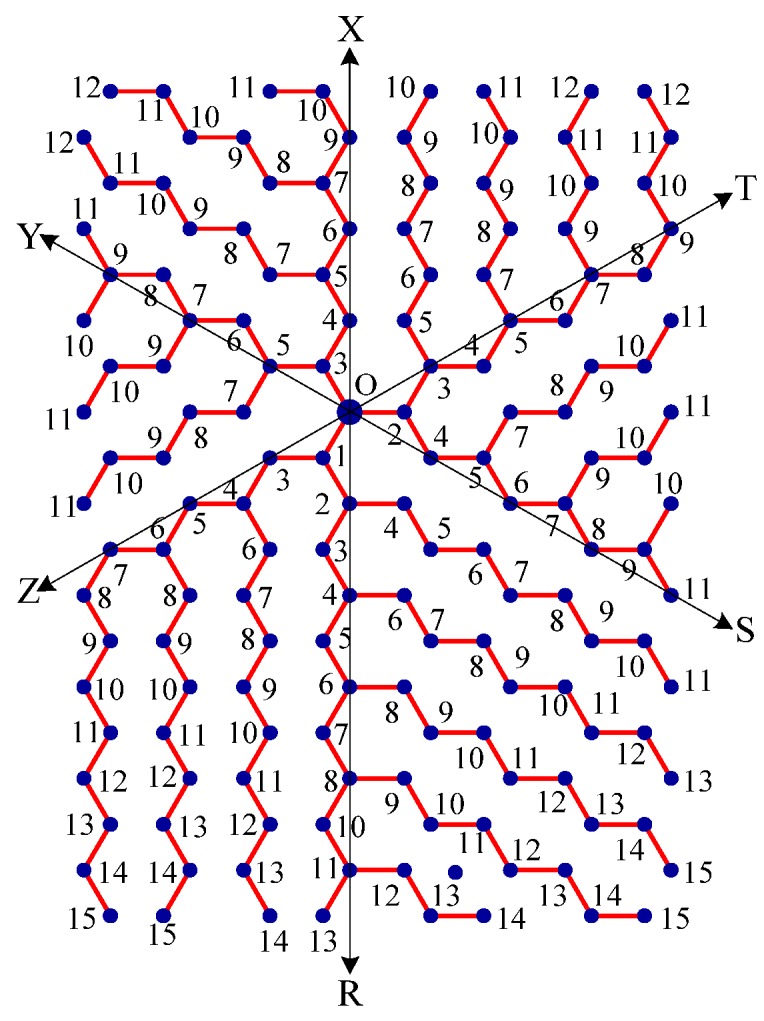
A node of label 5 means that the node receives the message from its neighbor at 5^th^ time unit.

Step 1: Let O be the source node with the message and E denote the eccentric node of O. Unfold the carbon hexagonal nanotube into a rectangular sheet in such a way that the eccentric vertex *E* lies on the perimeter of the rectangular sheet. See Figure 6.
Step 2: Draw lines TOZ (at angle 30°), ROX (vertical), and SOY (at angle 150°). These lines create six zones, namely, zones ROS, SOT, TOX, XOY, YOZ and ZOR. See Figure 7.
Step 3: Delete all the edges of zone ROS which are perpendicular to OS. Similarly, delete all the edges perpendicular to OT in zone SOT, edges perpendicular to OX in zone TOX, edges perpendicular to OY in zone XOY, edges perpendicular to OZ in zone YOZ, edges perpendicular to OR in zone ZOR. The resulting tree is the *broadcasting tree* of the carbon hexagonal nanotubes. See Figure 8.
Step 4: Message is disseminated from source O based on *farthest-distance-first protocol* where a node with the message chooses an uninformed adjacent node which leads to longest path in the tree. If a node has label *i*, it means that the node receives the message from its neighbor at *i*^th^ time unit. See Figure 9.

**Proof of Correctness**: Now we discuss the proof of correctness of the above algorithm. Let us recall that O is the source node, E is the eccentric vertex of O and *℮* is the shortest distance between O and E.

**Lemma 5.1**: The broadcasting algorithm for carbon hexagonal nanotubes delivers the message from source node O to all other nodes in *℮*+2 time units.

**Proof**: The path between a node *u* and O in the broadcasting tree is a shortest path between *u* and O in the carbon hexagonal nanotube [31]. Since the message is disseminated from one node to another node based on farthest-distance-first protocol, the node E receives the message in *℮* time units. Let us assume that E lies in zone ROS. By construction, the subgraph in zone ROS is a collection of paths parallel to the line OS. Thus by the time the message is delivered at E, all the nodes in zone ROS receive the message. Since the degree of O is 3, the other zones receive the message with the delay of 2 time units from O. However the distance of a node of other zones from O is less than *℮*. Using the above logic, the nodes of other zones receive the message in *℮*+2 time units.

**Theorem 5.2**: The broadcasting algorithm for carbon hexagonal nanotubes is optimal.

**Proof**: Since the degree of O is 3 and the eccentricity of source node O is *℮*, the minimum broadcasting time is *℮*+2. By Lemma 5.1, all the nodes of the carbon nanotube receive the message in *℮*+2 time units. Thus the algorithm is optimal.  ☐

### 5.2. Broadcasting Problem of Boron Triangular Nanotubes

In this section, we illustrate that the underlying broadcasting trees of carbon and boron nanotubes are similar. Here is the broadcasting algorithm of boron triangular nanotube.

**Figure 10:**
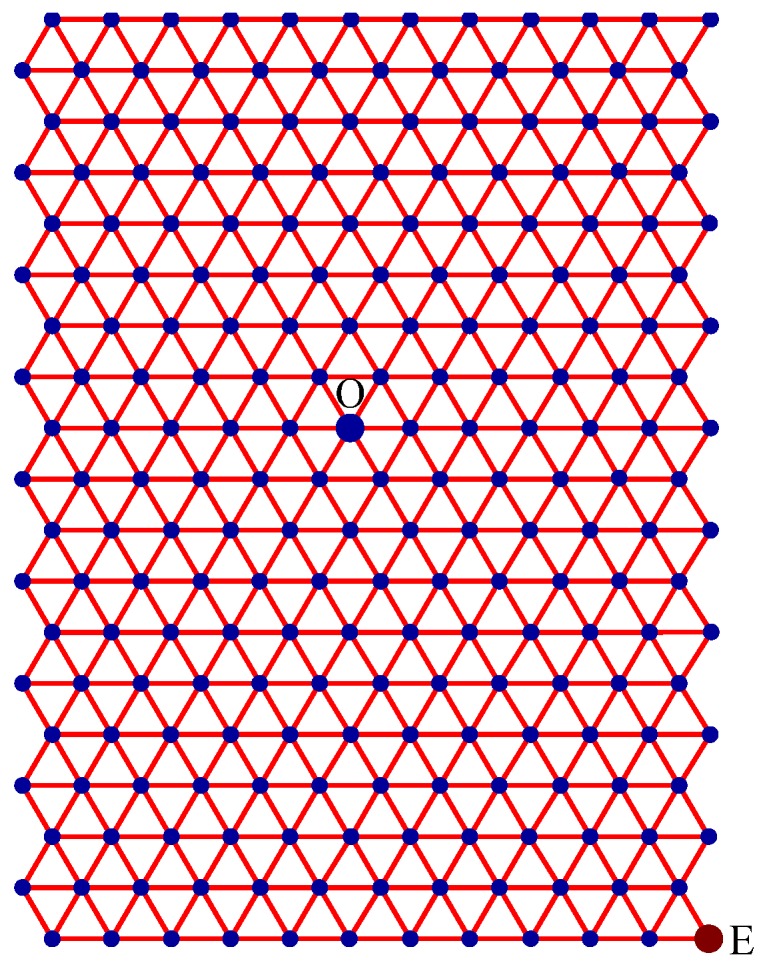
Node O is the source and node E is the eccentric node of O. The wrapping edges between the first column and the last column are not drawn.

**Figure 11:**
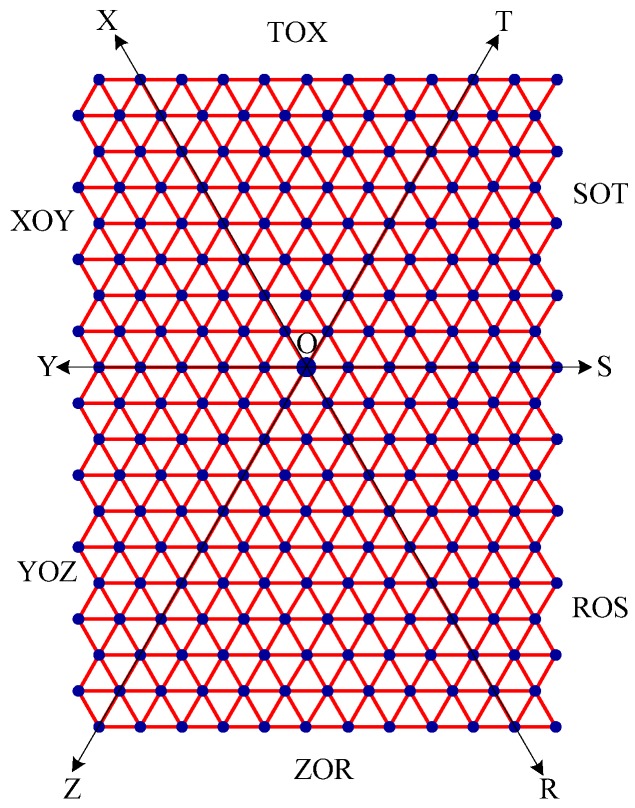
The rectangular sheet is divided into 6 zones: zones ROS, SOT, TOX, XOY, YOZ and ZOR.

**Figure 12:**
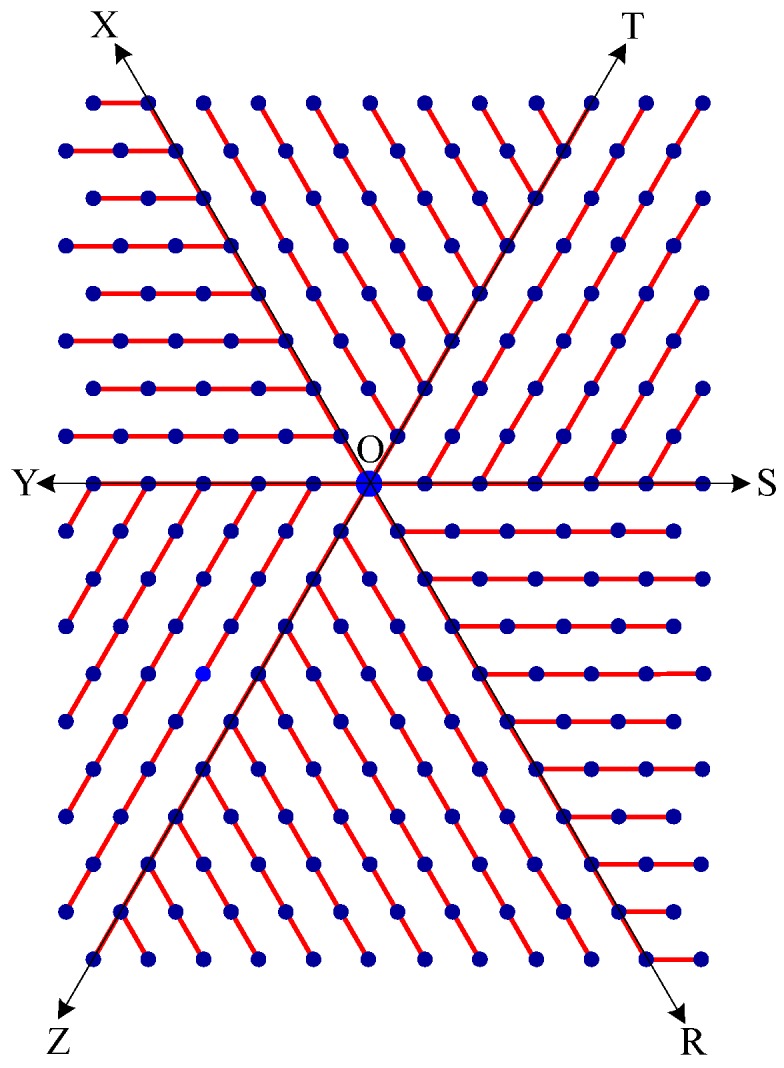
Broadcasting tree of boron nanotube. Broadcasting is based on farthest-distance-first protocol.

**Figure 13:**
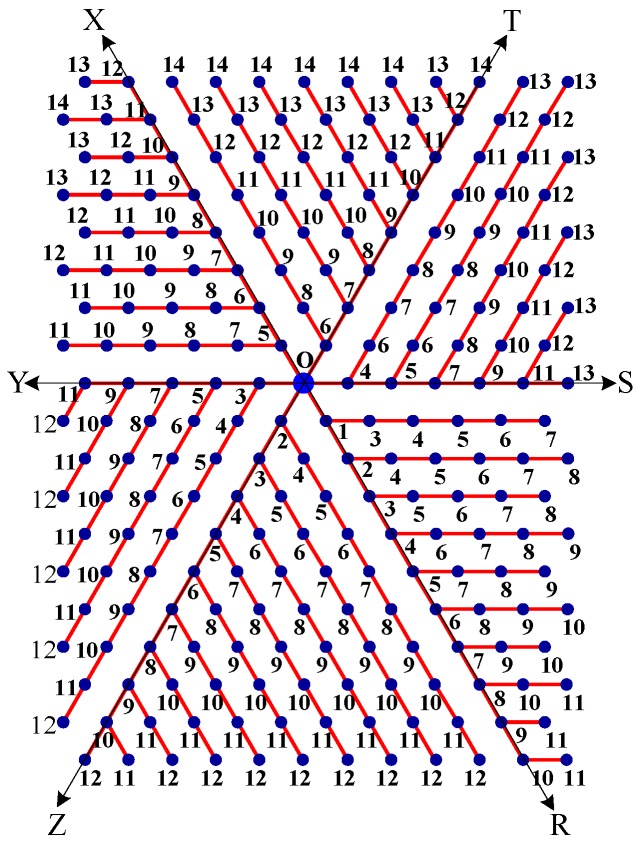
A node of label 5 means that the node receives the message from its neighbor at 5^th^ time unit.

Step 1: Let O be the source node with the message and E denote the eccentric node of O. Unfold the boron triangular nanotube into a rectangular sheet in such a way that the eccentric vertex E lies on the perimeter of the rectangular sheet. See Figure 10.
Step 2: Draw lines SOY (horizontal), TOZ (at angle 60°), and XOR (at angle 120°). These lines create six zones, namely, zones ROS, SOT, TOX, XOY, YOZ and ZOR. See Figure 11.
Step 3: Delete all the edges of zone ROS except the edges parallel to OS. Similarly retain only the edges parallel to OT in zone SOT, edges parallel to OX in zone TOX, edges parallel to OY in zone XOY, edges parallel to OZ in zone YOZ, and edges parallel to OR in zone ZOR. The resulting tree is the *broadcasting tree* of the boron triangular nanotube. See Figure 12.
Step 4: The message is disseminated from source O based on *farthest-distance-first protocol* where a node with the message chooses an uninformed adjacent node which leads to longest path in the tree. If a node has label *i*, it means that the node receives the message from its neighbor at *i*^th^ time unit. See Figure 13.

The proof of correctness of the algorithm is similar to the previous one. The degree of source O is 6 and ℮ is the eccentricity of O. Thus minimum broadcasting time to broadcast a message from O to all other nodes is ℮+5 because one zone receives the message from O with the time delay of 5 time units. Our algorithm broadcasts the message to all nodes in ℮+5 time units. Thus we state that

**Theorem 5.3**: The broadcasting algorithm for boron triangular nanotubes is optimal.

Broadcasting algorithm for boron α-nanotube is similar. We conclude in this section that all three nanotubes communicate in an identical fashion and have identical broadcasting spanning tree. 

## Conclusions

We have shown that the three nanotubes have the same maximum independent set and their matching ratios are independent of the number of columns. In addition, we have illustrated that they also have common underlying broadcasting spanning tree and identical communication behavior. It is interesting to explore further the similar nature of these nanostructures.
